# Direct observation of oxygen-vacancy formation and structural changes in Bi_2_WO_6_ nanoflakes induced by electron irradiation

**DOI:** 10.3762/bjnano.10.141

**Published:** 2019-07-18

**Authors:** Hong-long Shi, Bin Zou, Zi-an Li, Min-ting Luo, Wen-zhong Wang

**Affiliations:** 1 School of Science, Minzu University of China, Beijing 100081, People’s Republic of China, Tel. +861068930809; 2 Beijing National Laboratory for Condensed Matter Physics, Institute of Physics, Chinese Academy of Sciences, Beijing 100190, People’s Republic of China, Tel. +861082648001; 3 The National Engineering Laboratory for Hydrometallurgical Cleaner Production Technology, Institute of Process Engineering, Chinese Academy of Sciences, Beijing 100190, People’s Republic of China, Tel. +861082544809

**Keywords:** bismuth tungsten oxide, electron diffraction, electron irradiation, nanoflakes, oxygen vacancies

## Abstract

The prominent role of oxygen vacancies in the photocatalytic performance of bismuth tungsten oxides is well recognized, while the underlying formation mechanisms remain poorly understood. Here, we use the transmission electron microscopy to investigate the formation of oxygen vacancies and the structural evolution of Bi_2_WO_6_ under in situ electron irradiation. Our experimental results reveal that under 200 keV electron irradiation, the breaking of relatively weak Bi–O bonds leads to the formation of oxygen vacancies in Bi_2_WO_6_. With prolonged electron irradiation, the reduced Bi cations tend to form Bi clusters on the nanoflake surfaces, and the oxygen atoms are released from the nanoflakes, while the W–O networks reconstruct to form WO_3_. A possible mechanism that accounts for the observed processes of Bi cluster formation and oxygen release under energetic electron irradiation is also discussed.

## Introduction

Bi_2_WO_6_ has drawn great interest regarding its physical properties such as the piezoelectric effect and ferroelectricity with large spontaneous polarization and high Curie temperature [1–3], and pyroelectric and non-linear optical properties [4–5]. Recently, Bi_2_WO_6_ has shown good performance in the degradation of organic compounds [6–8], and photocatalytic oxygen evolution [9–10] and CO_2_ reduction [11–13] under visible-light irradiation. Bi_2_WO_6_ is the simplest member of the Aurivillius phases, perovskites with the general formula of (Bi_2_O_2_)(A*_m_*_−1_B*_m_*O_3_*_m_*_+1_) and a crystal structure of tilted WO_6_ octahedra layers sandwiched between Bi–O layers. The W–O layers in the Bi_2_WO_6_ crystal transfer electrons to the surface of catalysts, and Bi–O layers act as insulating layers that self-adapt to keep the balance of space charges.

Previous reports [14–15] have indicated that defects in Bi_2_WO_6_ affect its physical properties because defects can modify the band structure and electron–hole pairs [16–17]. Oxygen vacancies in the insulating layers of Bi_2_WO_6_ are defects that can be induced by chemical doping [18–19], hydrogen reduction [16] or ultra-thinning [14,20]. Surface oxygen vacancies can efficiently separate photogenerated electron–hole pairs, resulting in enhanced photocatalytic activity. Bismuth defects or dangling bonds of bismuth atoms resulting from oxygen vacancies can significantly alter the electron structure by creating new defect levels in forbidden bands, and thus boost oxygen evolution [14]. Despite the important role of the oxygen vacancies in Bi_2_WO_6_, understanding their formation mechanism remains elusive, in part due to the lack of direct observations of the oxygen-vacancy formation by in situ spectroscopy and microscopy methods. Therefore, it is desirable to perform in situ microscopy experiments to investigate the generation and evolution of oxygen vacancies at the insulating layer of Bi_2_WO_6_ crystals upon an external stimulation, which will be a fundamental step for controllable engineering of defects.

Electron-beam irradiation is a powerful technique to fabricate or modify materials at the nanoscale [21–22]. For example, electron irradiation can induce a phase transformation from crystalline to amorphous or vice versa [23–24]; the knock-on effect of electron-beam irradiation will break chemical bonds and or knock off atoms directly from the surface of the irradiated material. Generally, it is thought that electron irradiation of materials containing heavy atoms by using a traditional transmission electron microscope (TEM) is not effective. Considering that the atomic escape energies are relatively small [25–26], Bi_2_WO_6_ nanoflakes with a thickness below 10 nm will be favorable when defects are to be induced through electron irradiation. When subjected to intense electron-beam irradiation, various types of crystal defects, including oxygen vacancies and or bismuth defects, can be generated in Bi_2_WO_6_ nanoflakes and simultaneously be observed by in situ TEM.

In this study, Bi_2_WO_6_ nanoflowers aggregated from numerous nanoflakes of ca. 10 nm in thickness and ca. 100 nm in flake size, were synthesized by a solvothermal method. A series of high-resolution TEM (HRTEM) imaging and electron diffraction experiments was performed to investigate the electron-induced defects in the Bi_2_WO_6_ nanoflakes. Our results reveal that Bi_2_WO_6_ nanoflakes can be decomposed into Bi precipitates and WO_3_ nanosheets after the generation of oxygen vacancies during the electron-beam irradiation process. The formation mechanisms of Bi/O defects are discussed in detail by combining the HRTEM imaging of defects and the calculation of the electrostatic site potentials of Bi_2_WO_6_.

## Results and Discussion

### Structural features and photodegradation

**Figure 1:**
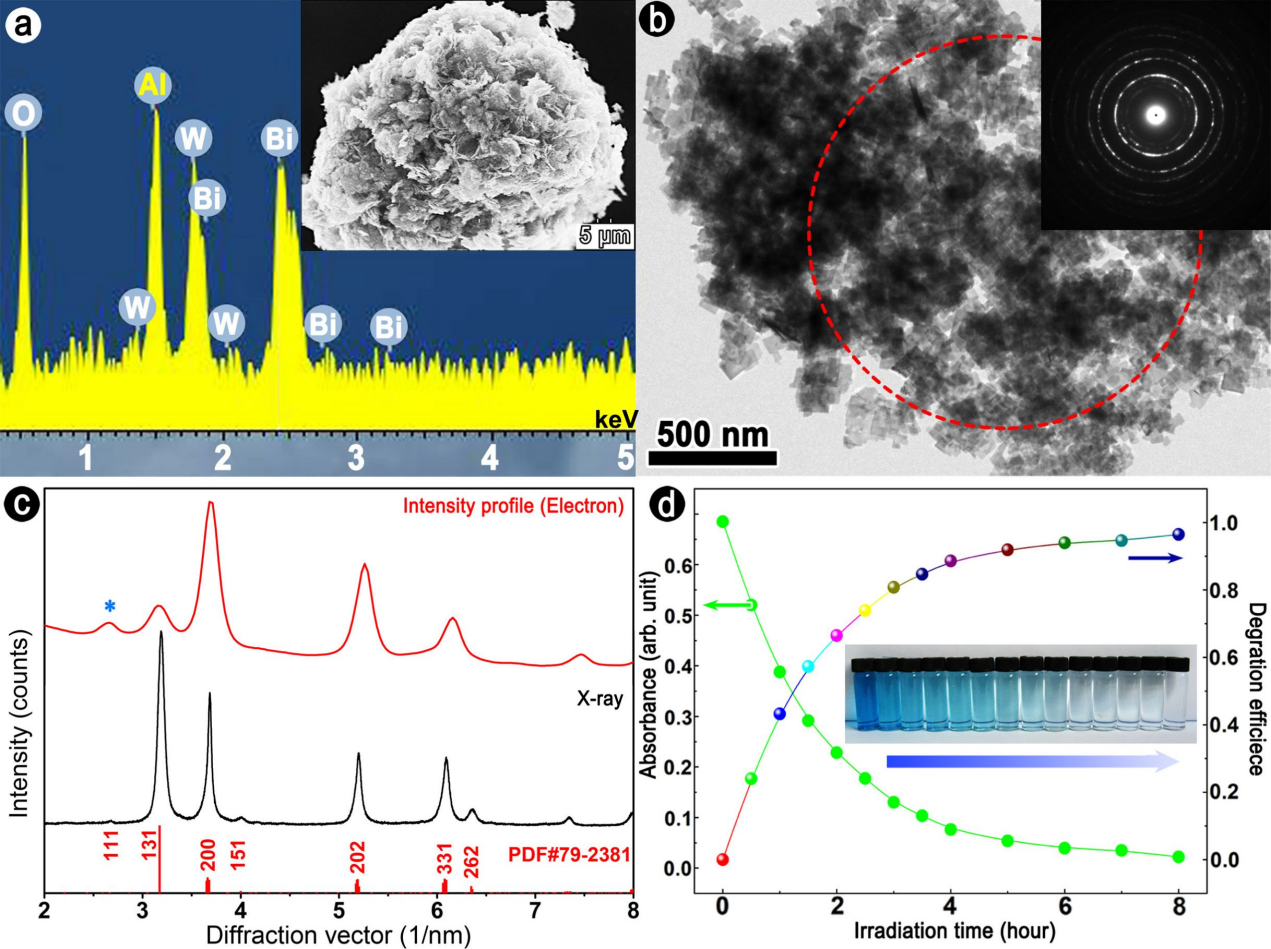
(a) Energy-dispersive X-ray spectrum of Bi_2_WO_6_ nanoflowers, the inset is a typical SEM image; (b) TEM bright-field image and SAED pattern from the analysis region marked by the red dashed circle; (c) XRD pattern (black) and intensity profile (red) of the SAED pattern, overlaid by a standard PDF card; (d) Absorbance and degradation efficiency as a function of irradiation time, the inset is a photograph of the degradation experiment.

The Bi_2_WO_6_ sample is a fine white powder consisting of flower-like aggregates (see the SEM image in the inset of Figure 1a). The typical size of the aggregates is about 20–30 μm. Energy-dispersive X-ray (EDX) spectroscopy indicates the presence of Bi, W, and O in these aggregates with the atomic ratio of ca. 2:1:6. Note that the Al peak located at 1.5 keV is from the sample holder. Figure 1b shows a low-magnification TEM image of the specimen, illustrating that these aggregates are composed of crystalline nanoflakes with sizes of 50–100 nm. The thickness of these nanoflakes is 6–14 nm, as determined by HRTEM simulations based on the Bloch wave method [27].

Both X-ray diffraction pattern (XRD) and intensity profile of the SAED (Figure 1c) reveal that the as-synthesized sample crystallizes into an orthorhombic russellite phase (PDF#79-2381) with space group *Pca*2_1_ (no. 29). The refined lattice parameters are *a* = 5.4063(4) Å, *b* = 16.4186(0) Å, and *c* = 5.4319(7) Å. An extraordinary strong (200) reflection in the SAED pattern indicates the strong preferred orientation of the flake-like nanocrystallites. This effect is further enhanced in TEM experiments because the ultrasonic treatment in absolute ethyl alcohol during the TEM specimen preparation will make the flower-like aggregates disperse into individual nanoflakes perpendicular to the incident electron beam. It is noteworthy that, compared with XRD, an additional peak or diffraction spot located at 2.654 nm^−1^ (or *d* = 3.768 Å, marked with the asterisk in Figure 1c) was frequently encountered and its intensity is enhanced during the routine TEM observation, accompanied by the appearance of dark precipitates on the surface of nanoflakes. This suggests that the as-synthesized Bi_2_WO_6_ nanoflakes are sensitive to the electron beam.

The photocatalytic activity of the sample was measured by the degradation of methylene blue under visible-light irradiation using a 300W Xe lamp, which is shown in Figure 1d. After irradiation for 3 h, the degradation efficiency of the sample increases to over 80%. This may result from the high concentration of surface dangling bonds of these thin nanoflakes [14].

### Electron irradiation of an individual nanoflake through HRTEM

In order to avoid secondary electron scattering and moiré fringes in HRTEM imaging, we selected a well-isolated individual nanoflake of ca. 70 nm in size for electron irradiation experiments. The size of the incident electron beam was tuned to match the size of the irradiated nanoflake, and the beam current was measured to be about 40 pA/cm^2^.

**Figure 2:**
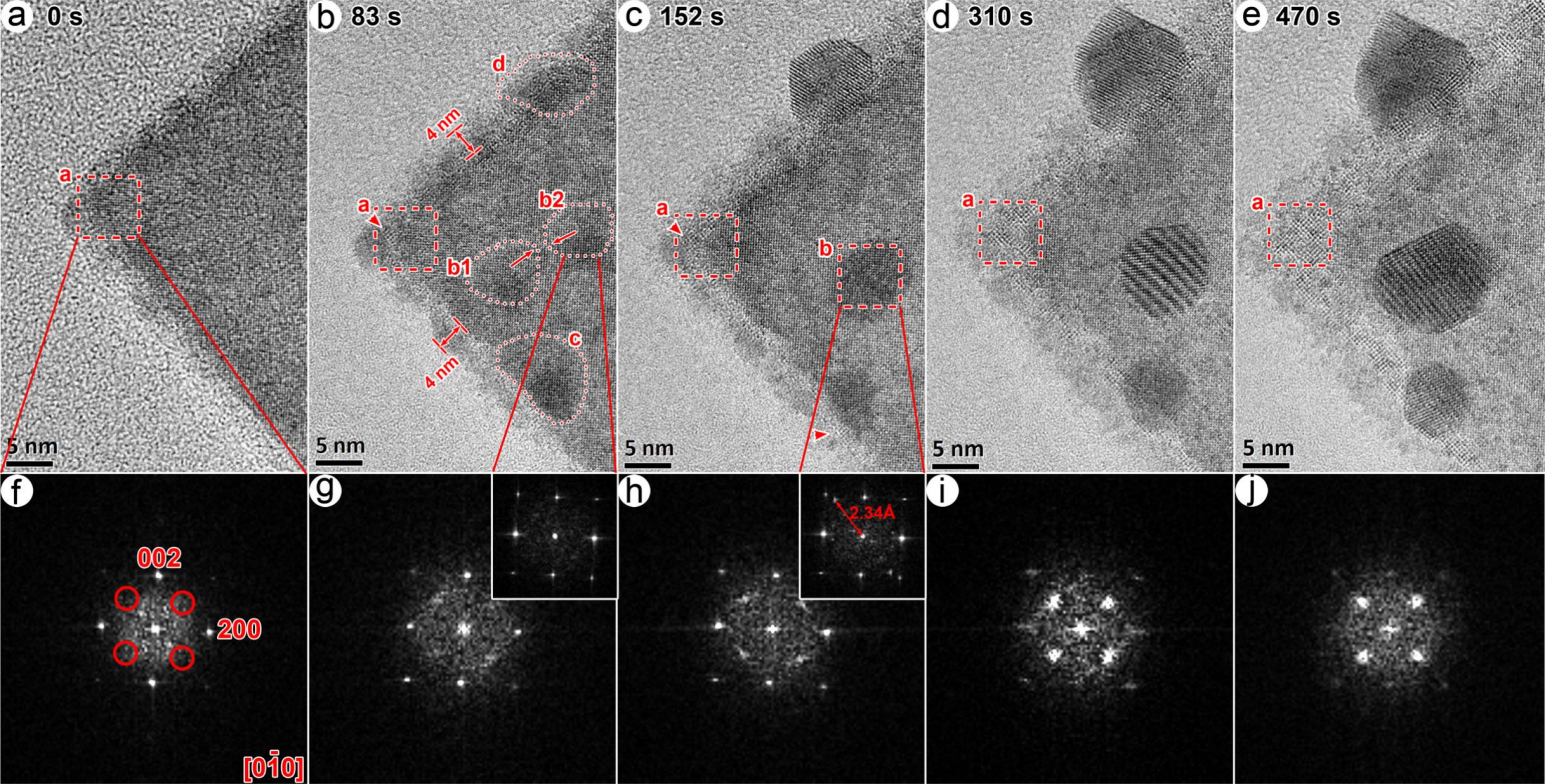
Effects of electron-beam irradiation on an individual Bi_2_WO_6_ nanoflake observed by using HRTEM. (a–e) Sequential HRTEM images, and (f–j) the corresponding diffractograms calculated from region “a”. The two insets in Figure 2g,h are calculated from the regions “b2” and “b”, respectively. The red circles in the diffractogram of Figure 2f indicate the extinction positions of the Bi_2_WO_6_ structure.

Figure 2 shows a series of HRTEM images of an individual Bi_2_WO_6_ nanoflake irradiated by a high-energy electron beam (working at 200 kV) and the corresponding FFT diffractograms from the region marked as “a” in Figure 2a. Before irradiation (Figure 2a), it was a quadrangular flake with a smooth surface exhibiting no contrast variation. Also, there were no detectable diffraction spots at the extinction positions (101) marked by the red circles in Figure 2f. After irradiation for 83 s (Figure 2b), four bubble-like structures (ca. 5–8 nm in size) with a dark contrast (labeled as “b1”, “b2”, “c” and “d”) appeared on the surface of the examined nanoflake. It is worthy of note that there was still no detectable difference in the diffractograms of the bubbles (inset of Figure 2g) and the initial flake (Figure 2f), but some weak scattering appeared at the extinction positions (Figure 2g) in the diffractogram of region “a”. In other words, at this stage the bubbles possess a structure similar to that of the parent phase. Moreover, this flake was coated by a ca. 4 nm thin amorphous layer. These observed features on the Bi_2_WO_6_ flake indicate that bonds were broken in the nanoflake, and atoms were expelled towards the surface of the irradiated flake by the released gas and/or the electron-beam-induced electric field [28–29].

The bubbles appeared to be mobile, and the bubbles marked by “b1” and “b2” merged to form a larger one, marked as “b”, after irradiation for 152 s (Figure 2c). The merged bubble possessed additional diffraction spots with *d* ≈ 2.34 Å (inset of Figure 2h). Bubble “d” crystalized and exhibited clear lattice fringes with *d* ≈ 3.27 Å. These result indicate that the released atoms tend to aggregate on the surface of the flakes, and then recrystallize into new crystallites. Moreover, larger lattice spacings (*d* ≈ 3.768 Å, arrows indicated in Figure 2c) were observed at the edges of the irradiated flake, resulting in stronger electron scattering at the extinction positions in the diffractogram of region “a” (Figure 2h). After irradiation times of 310 s (Figure 2d) and 470 s (Figure 2e), precipitates grew at the nanoflake surface at the expense of the initial Bi_2_WO_6_. This process is also clearly reflected in the diffractograms in Figure 2i and Figure 2j in which the diffraction spots of Bi_2_WO_6_ gradually disappeared, and spots emerge at the extinction positions. Eventually, the precipitates grew up to 10–20 nm in size while the degraded amorphous layers become thinner and more uniform.

### Structural analysis by in situ SAED and HRTEM

**Figure 3:**
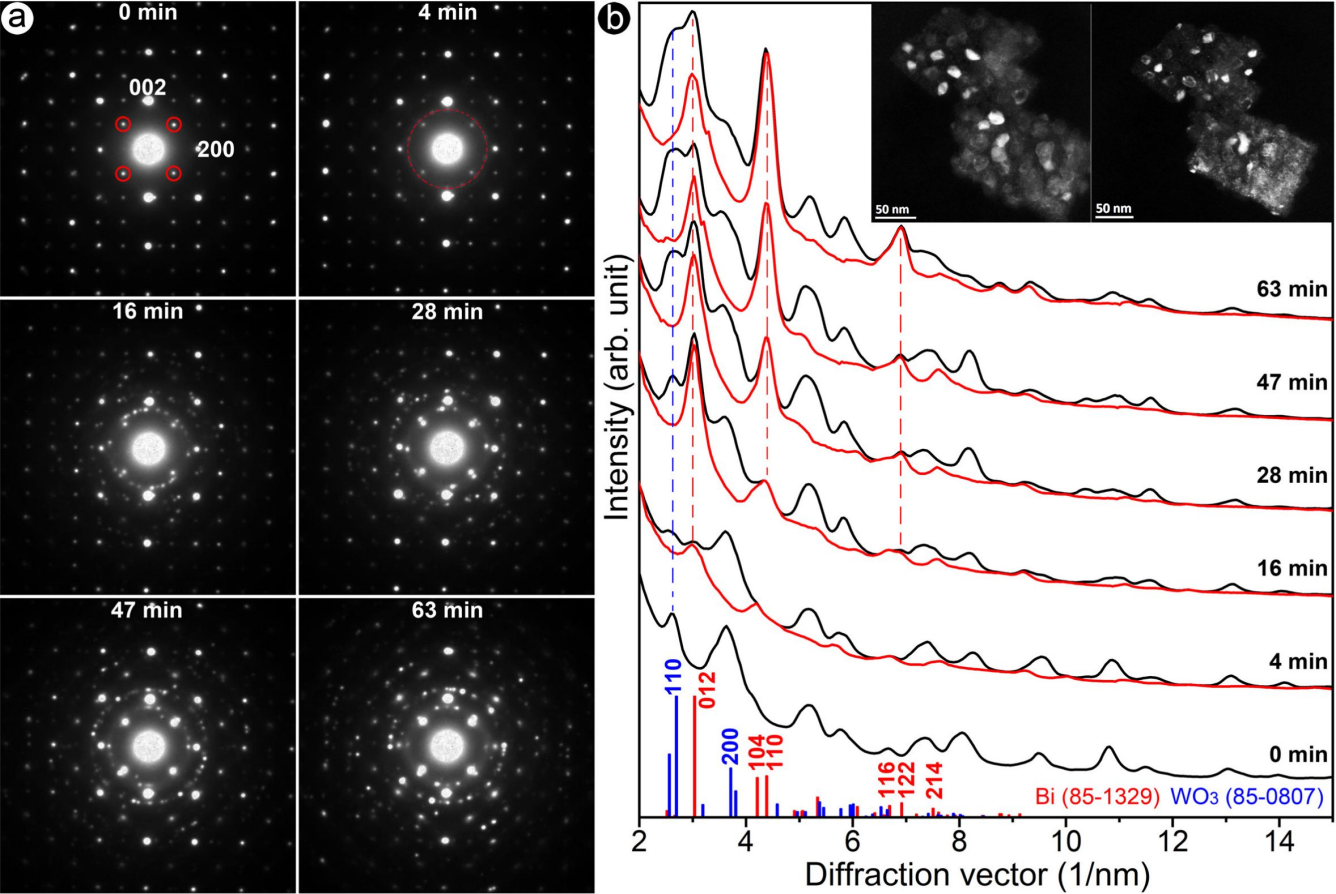
Effects of the irradaition on Bi_2_WO_6_ investigated by SAED. (a) A series of SAED patterns after irradiation for 0, 4, 16, 28, 47 and 63 min, and (b) the corresponding intensity profiles. The black curves are the total diffraction data and the red curves are the residual scattering intensities after filtering the {101} scattering at the extinction positions of Bi_2_WO_6_ (red circles in Figure 3a). The red and blue bars are standard peaks for Bi (PDF#85-1329) and WO_3_ (PDF#85-0807). The inset shows two typical dark-field images during the electron irradiation.

The above HRTEM experiments reveal the structural evolution of Bi_2_WO_6_ nanoflakes under relatively strong electron irradiation. In order to determine the resultant crystallographic structures of the precipitates and the remaining layers, a second in situ TEM experiment was designed and carried out. The sample was a flat complex (inset of Figure 3b) consisting of two large nanoflakes each of ca. 140 nm in size, and the specimen was tilted along the [010] zone axis. In order to capture the transition state, the electron beam was spread out to match the large screen of the TEM to reduce the electron dose, and the exposure time was 2.5 s to record the SAED patterns. In order to conveniently perform a “search–match” analysis, the intensity profiles of the precipitates (red curves in Figure 3b) are extracted by the rotation–averaging process [30] of SAED patterns after filtering the {101}_Bi2WO6_ scattering signals by adding an array mask. The masked pattern represents the filtered phase and the unmasked pattern represents the precipitates.

**Figure 4:**
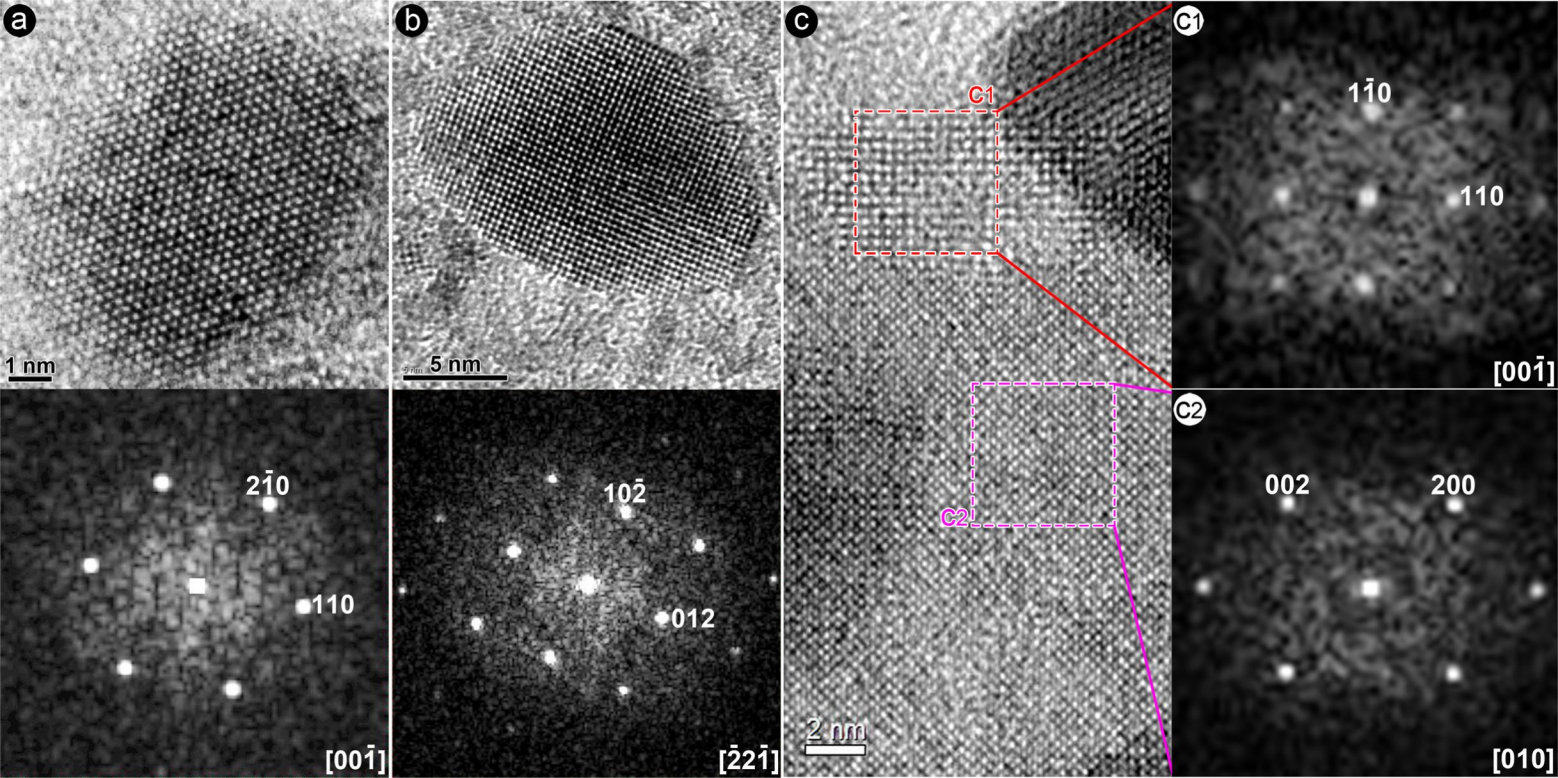
HRTEM analysis of the electron-beam-induced products: (a, b) two typical HRTEM images of precipitates and the corresponding diffractograms; (c) a typical HRTEM image for the coexistence of domains and the parent phase, and (c1, c2) the corresponding diffractograms.

Figure 3 shows a series of SAED patterns recorded after electron irradiation for up to 62 min. Note that prior to irradiation some very weak scattering signals (red circles in Figure 3a) are present, which arise from the secondary electron scattering of the two nanoflakes. After irradiation for 4 min, these weak scattering signals were significantly weakened while a weak spotty ring appeared at ca. 3.02 nm^−1^. For the precipitates (red curves in Figure 3b), diffraction peaks appeared at 3.02, 4.39, 6.68, 7.61 nm^−1^, which are in good agreement with the (012), (104), (116) and (214) planes of the hexagonal bismuth phase (PDF#85-1329). Moreover, no reflections that can be associated with the crystal structure of bismuth oxides appear during the whole irradiation process. This suggests that released Bi and O do not recrystallize into BiO*_x_*; instead the Bi atoms precipitate at the surfaces to form Bi clusters, and the O atoms are released into the vacuum. The background-removed intensity ratio *I*_(110)_/*I*_(012)_ of bismuth precipitates was 0.68, 0.35, 0.83, 1.42, and 1.84 after an irradiation time of 4, 16, 28, 47 and 63 min, hinting at the variation of the preferred growth orientation of bismuth crystallites on the surface of the Bi_2_WO_6_ nanoflake. Analysis of HRTEM images of precipitates (Figure 4a,b) confirms the formation of a hexagonal bismuth phase. The zone-axis directions of the corresponding diffractograms are 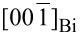 and 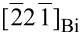.

Phase identification of domains with wide lattice fringes using the traditional search–match method remains difficult, because the scattering signals of the domains just occur at those of the parent phase (or its extinction positions). For the region with wide lattice fringes, we therefore determine the unit cell by combining the scattering signals and their respective symmetry in the diffractogram. Figure 4c shows an HRTEM image for the coexistence of the parent phase labeled “c2” and the phase with wide lattice fringe labeled “c1”. The square-like diffractogram of the wide lattice fringes in Figure 4c1 suggests that it crystallized in a cubic or a tetragonal phase. The diffraction peak at 3.738 Å was constrained as (110) during the indexing process. Six diffraction peaks extracted from its diffractogram pattern (Table 1) were used to perform ab initio indexing, revealing that the region with wide lattice fringes can be indexed to tetragonal tungsten oxide with lattice parameters of *a* = *b* = 5.2692(5) Å, and *c* = 3.9588(6) Å. Thus, the zone axis of the diffractogram in Figure 3c1 is 
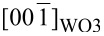
.

**Table 1:** List of diffraction peaks used to perform ab initio indexing of the wide lattice fringes. Note: the characteristic diffraction spot at 3.738 Å was constrained to (110) in the pattern indexing based on the symmetry of Figure 4c1.

*d*-spacing	(*hkl*)

3.738 Å	(110)
2.629 Å	(200)
1.869 Å	(220)
1.692 Å	(310)
1.315 Å	(400)
1.246 Å	(330)

The above crystal phase analysis indicates that under electron-beam irradiation Bi_2_WO_6_ nanoflakes decompose into three phases: (1) hexagonal bismuth precipitate, (2) tetragonal tungsten oxide, and (3) the remaining defective Bi_2_WO_6_:


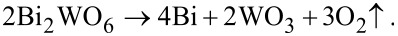


The released oxygen atoms are removed from Bi_2_WO_6_ and oxygen vacancies are thus introduced into Bi_2_WO_6_ crystals. Alternatively, the oxygen vacancies can be also generated by hydrogen reduction at a temperature range from 150 to 275 °C for 5 h [16].

### Formation of oxygen vacancies and other defects in Bi_2_WO_6_

**Figure 5:**
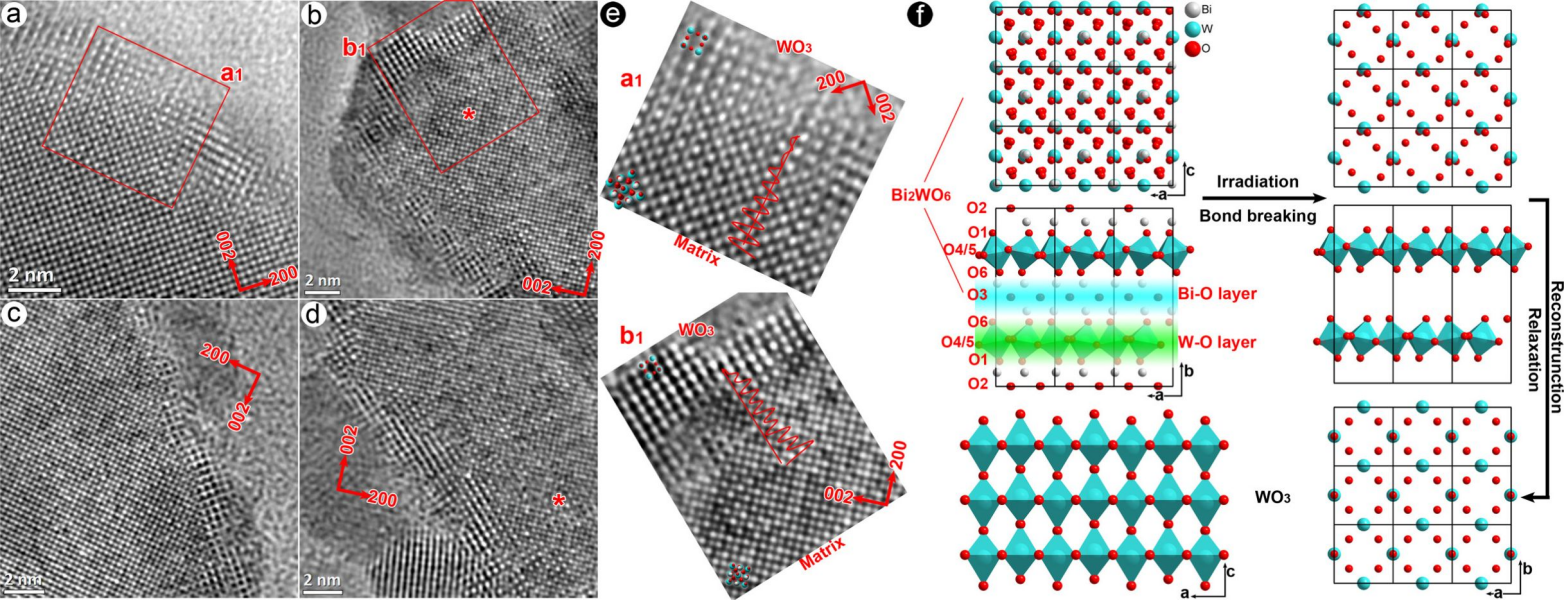
Formation of oxygen vacancies in Bi_2_WO_6_ nanoflakes through electron irradiation: (a–d) four typical HRTEM images of the irradiated specimens; (e) enlarged views of regions a1 and b1, where contrast variations (red curves) of atom columns pointing to the surface are overlaid to indicate the decrease of the Bi/O atom concentration; and (f) a schematic plot of the structure transformation from Bi_2_WO_6_ to WO_3_: breaking of bonds in the Bi–O layers induced by electron-beam irradiation, relaxation of W–O layers and reconstruction of the WO_3_ phase.

The above in situ experiments indicate that oxygen vacancies can be effectively produced by electron-beam irradiation. Thus irradiation in TEM will be an alternative method to generate oxygen vacancies in the thin Bi_2_WO_6_ nanoflakes. In order to demonstrate the formation of vacancies in Bi_2_WO_6_ nanoflakes, Figure 5a–d shows four typical HRTEM images after electron-beam irradiation. The interior region (the parent phase) of nanoflakes shows a uniform contrast of the atom columns indicating the complete occupancy of Bi, W and O atoms in the interior region. Figure 5e shows a large view of regions “a1” and “b1” in Figure 5a and Figure 5b. The contrast of atom columns (or the overlaid intensity profiles) along the ⟨101⟩_Bi2WO6_ direction pointing to the outside of the nanoflake is evidently decreased, resulting from a loss of Bi and/or O atoms during irradiation. At the periphery of the irradiated nanoflakes (WO_3_ phase), there are some regular, wide lattices without the lattice center, indicating that the residual WO_6_ octahedra have been completely transformed into WO_3_. In contrast, in the transition regions marked with the asterisk in Figure 5b and Figure 5d, 3–5 nm from the surface, there are still some wide lattice fringes but with center atoms, implying only a few Bi–O bonds were broken and only a part of the residual WO_6_ octahedra transformed into WO_3_. These features indicate bond breaking within Bi–O layers and the reconstruction of WO_6_ octahedra predominantly occur at the surfaces of the irradiated nanoflake.

In general, electron irradiation of materials results in three major effects: heating, sputtering and radiolysis. The maximum temperature increase through electron-beam irradiation was calculated according to the following formula [31]:

[1] $$\Delta T=\frac{1}{4\pi ke}\frac{\Delta E}{d}\left(1+\ln\frac{b}{r_{0}}\right)\;,$$

where *I*, *k*, *e*, *b*, *r*_0_, and Δ*E* are the beam current, thermal conductivity, electron charge, sample radius, beam radius, and the total energy loss per electron in a sample of thickness *d*, respectively. For the irradiation experiment of an individual nanoflake described above, the incident beam size is matched with the irradiated specimen, and the maximum temperature increase is ca 1.2 °C when assuming *d* = 10 nm, and *k* = 2.65 W·K^−1^·m^−1^ [32]. Apparently, the heating effect of the electron-beam irradiation, in this case, is far too low to decompose Bi_2_WO_6_ nanoflakes. Besides, loops or defect clusters were not observed during the whole irradiation process suggesting that the knock-off or sputtering effects can be neglected.

When a high-energy electron beam passes through the sample, an electric field will be induced by the accumulation of secondary electrons and Auger electrons in the irradiation region [28–29]. The charged ions in the crystal will be displaced, and ionic bonds can be broken when the induced electric field, which can be enhanced with the increase of the irradiation time, is stronger than a threshold field, which mainly depends on bond strength or electrostatic site potential of atoms in the crystal.

Table 2 lists the electrostatic site potentials of Bi_2_WO_6_ calculated by using the VESTA software based on the Fourier method [33]. The oxygen atoms labeled “O4” and “O5” (Figure 5f) with large potentials (2.2317 and 2.2967 e/Å) are strongly bonded with tungsten atoms, generating stable WO_6_ octahedra; while oxygen atoms “O2” and “O3” possessing small site potentials (0.9992 and 1.0432 e/Å) form the weak chemical bonds “Bi1–O3” and “Bi2–O2”. Thin flakes (ca. 10 nm) can offer numerous surface active sites that facilitate the migration of ions. Therefore, upon electron-beam irradiation, Bi–O bonds will first break under the influence of the induced electric field. The resultant Bi cations migrate towards the periphery of the irradiation region to form a ca. 4 nm amorphous layer (Figure 2b), while the resultant O ions are retained in the irradiation region and subsequently released as O_2_ bubbles (“b1”, “b2”, “c” and “d” in Figure 2b). When only a small part of Bi–O bonds is broken, there will be no new electron scattering signals along the [010]_Bi2WO6_ zone axis. Only the overall scattered intensities will change because of symmetry constraints. Hence, there are no extra scattering signals at the extinction positions of the parent phase (inset of Figure 2g), although some dark bubbles were observed in Figure 2b. When the insulating layer is seriously damaged, the residual W–O layers will relax and then reconstruct as WO_3_, showing scattering signals at the extinction positions of the parent phase (Figure 2g) and generating the wide lattice fringes.

**Table 2:** The electrostatic site potentials of Bi_2_WO_6_ calculated via the Fourier method.

atoms	charge	atomic site	site potential (e/Å)

Bi1	+3	0.5206, 0.4224, 0.9761	−2.3009
Bi2	+3	0.4824, 0.0771, 0.9796	−2.3541
W1	+6	0.0071, 0.2495, 0.0000	−3.9664
O1	−2	0.0579, 0.1402, 0.0768	1.7113
O2	−2	0.2597, 0.9994, 0.2635	0.9992
O3	−2	0.2403, 0.5006, 0.2576	1.0432
O4	−2	0.7059, 0.2324, 0.2507	2.2317
O5	−2	0.2131, 0.2639, 0.3308	2.2967
O6	−2	0.5616, 0.3598, 0.5618	1.7367

Because the structural instability of the insulating layers of the Bi_2_WO_6_ crystal primarily depends on the site potential of O2 and O3 atoms, it is possible to controllably tune the concentration of oxygen vacancies by modifying the site potential of O2 and O3 through, e.g., fluorination or sulfidation of Bi_2_WO_6_.

## Conclusion

In summary, we have synthesized Bi_2_WO_6_ nanoflakes of 6–10 nm in thickness by using a solvothermal method, and then performed in situ electron irradiation experiments on the as-synthesized Bi_2_WO_6_ nanoflakes by using TEM techniques. We observed that 200 keV electrons are an efficient source of irradiation to induce defect states in thin Bi_2_WO_6_. Our detailed HRTEM and SAED analyses have revealed that energetic electrons break the relatively weak Bi–O bonds in Bi_2_WO_6_ crystals forming oxygen vacancies. With prolonged electron irradiation, Bi cations were found to be reduced to Bi atoms and to be expelled towards the sample surface to recrystallize, whereas oxygen atoms or molecules were released from the sample. Local electric fields contribute to the observed process of Bi cluster formation and oxygen release. Our experimental results suggest that the use of energetic electrons in a TEM is an effective means to induce oxygen vacancies or other defects in bismuth tungsten oxides. We suggest that it is possible to controllably tune the concentration of oxygen vacancies by modifying the site potential of O_2_ and O_3_ atoms via fluorination or sulfidation.

## Experimental

### Solvothermal synthesis

Bi_2_WO_6_ nanoflakes were synthesized by a solvothermal method. All chemical reagents were of analytical purity. Into 60 mL of deionized water, 0.97 g (0.002 mol) Bi(NO_3_)_3_·5H_2_O, 0.15 g cetyltrimethylammonium bromide (CTAB), and 0.33 g (0.001 mol) Na_2_WO_4_·2H_2_O were added successively under magnetic stirring at room temperature to yield the precursor suspension. The precursor suspension was further transferred into a Teflon-lined stainless autoclave (*V* = 100 mL). The autoclave was sealed and maintained at 180 °C for 12 h and then let to cool to room temperature. The resultant white powder was collected and washed with deionized water and absolute ethanol several times, and finally dried at 60 °C. The powder was dispersed in absolute ethanol and subsequently dripped onto conducting carbon resin for scanning electron microscopy (SEM) and onto carbon grids for TEM experiments.

### Microstructural characterization

X-ray diffraction experiments were performed on Bruker D8 Advance diffractometer using Cu Kα radiation (λ = 1.5406 Å). Morphological analyses were carried out on a field-emission scanning electron microscope (SEM, Hitachi S-4800) equipped with an energy-dispersive X-ray spectroscopy (EDX) detector operating at 10 kV and 10 μA. Selected-area electron diffraction (SAED) and high-resolution transmission electron microscopy (HRTEM) experiments were carried out on a JEM-2100 (JEOL Inc.), operating at 200 kV. Electron-beam irradiation experiments were performed by focusing the electron beam on the examined specimen regions. Typical experimental parameters were a current density of about 40 pA/cm^2^, and a C_2_ aperture (condenser lens aperture) opening of 70 μm.

### Experiments of photocatalytic activity

The photocatalytic activity of the samples was determined by measuring the degradation of methylene blue (MB) under visible-light irradiation using a 300 W Xe lamp with a 420 nm cut-off filter. In the experiment, 40 mg Bi_2_WO_6_ photocatalyst was dispersed into 100 mL of 10 mg/L MB solution. Then the suspensions were magnetically stirred for 1 h in the dark to ensure the establishment of an adsorption–desorption equilibrium between photocatalyst and MB. Then the solution was exposed to visible-light irradiation under magnetic stirring. At constant intervals of time, samples were taken from the suspension and centrifuged. Then the absorbance of the solution was analyzed at a wavelength of 664 nm. The photocatalytic efficiency of Bi_2_WO_6_ was taken calculated as follows: η(%) = (*A*_0_ – *A*)/*A*_0_·100%, where *A*_0_ and *A* are the absorbance of MB before and after the visible light irradiation, respectively.

## Supporting Information

File 1 Thickness determination via HRTEM simulation.
