# Supplementary material for: Direct observation of oxygen-vacancy formation and structural changes in Bi2WO6 nanoflakes induced by electron irradiation
Source: Beilstein J Nanotechnol. 2019 Jul 18;10:1434–42. doi: 10.3762/bjnano.10.141 (PMC6664412; doi:10.3762/bjnano.10.141)
Supplement: File 1 — Thickness determination via HRTEM simulation. [file Beilstein_J_Nanotechnol-10-1434-s001.pdf]

BEILSTEIN JOURNAL OF NANOTECHNOLOGY

Supporting Information

for

Direct observation of oxygen-vacancy formation and structural changes in Bi_2WO_6 nanoflakes induced by electron irradiation

Hong-long Shi, Bin Zou, Zi-an Li, Min-ting Luo and Wen-zhong Wang

Beilstein J. Nanotechnol. **2019,** *10,* 1434–1442. doi:10.3762/bjnano.10.141

Thickness determination via HRTEM simulation

License and Terms: This is a supporting information file under the terms of the Creative Commons Attribution License (http://creativecommons.org/licenses/by/4.0). Please note that the reuse, redistribution and reproduction in particular requires that the authors and source are credited.
The license is subject to the *Beilstein Journal of Nanotechnology* terms and conditions: (https://www.beilstein-journals.org/bjnano)

The thickness of the Bi_2WO_6 nanosheets was determined by HRTEM simulation based on the Bloch-wave method in the Jems software. The crystal structure is from ICSD-67647, the zone axis is [010]. The main simulation parameters were:

illumination model = envelop;

defocus min = 35 nm, step =1 nm, number = 10;

image dup-*x*/*y* = 2, noise% = 2,

start after = 3, number = 18, increment = 3 for iteration parameters

strong parameters = 25, Blochwave parameters: Bethe.

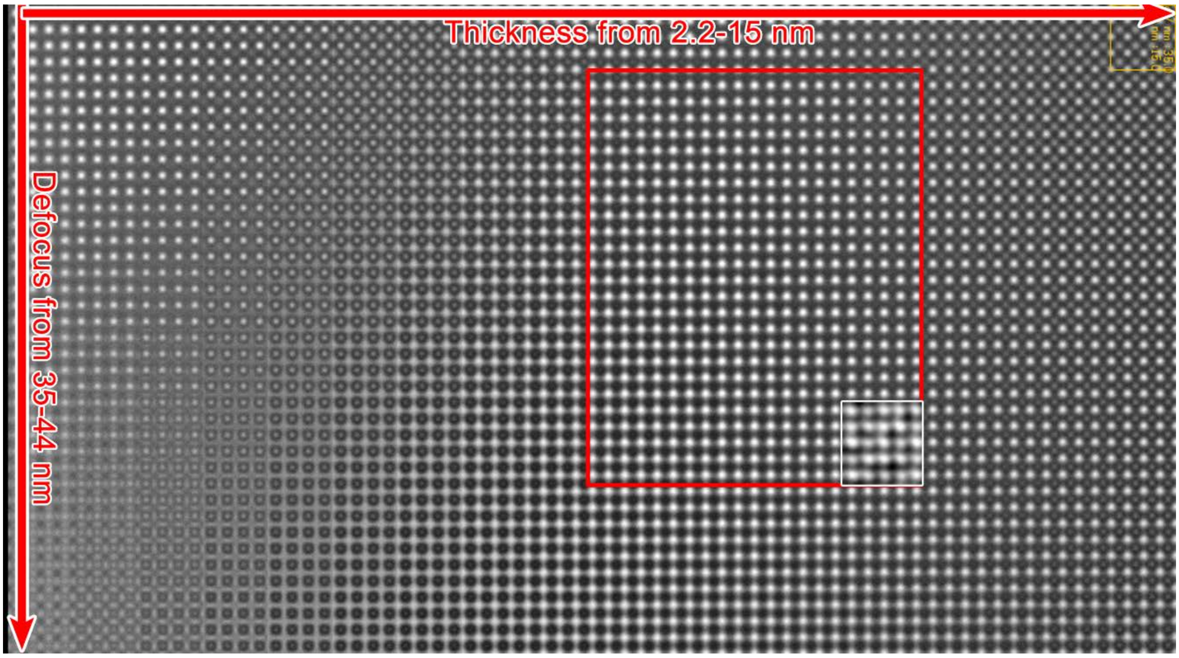

Figure S1: Thickness determination of the Bi_2WO_6 nanosheet by HRTEM image simulation, where the inset is the experimental HRTEM image, and the matched area is marked by a red frame. It indicates the thickness ranges of 6–14 nm.
